# Marijuana and Opioid Use during Pregnancy: Using Zebrafish to Gain Understanding of Congenital Anomalies Caused by Drug Exposure during Development

**DOI:** 10.3390/biomedicines8080279

**Published:** 2020-08-08

**Authors:** Swapnalee Sarmah, Marilia Ribeiro Sales Cadena, Pabyton Gonçalves Cadena, James A. Marrs

**Affiliations:** 1 Department of Biology, Indiana University Purdue University Indianapolis, 723 West Michigan St., Indianapolis, IN 46202, USA; 2 Departamento de Biologia (DB), Universidade Federal Rural de Pernambuco. Av. Dom Manoel de Medeiros s/n, 52171-900 Dois Irmãos, Recife - PE, Brasil; marilia.sales@ufrpe.br; 3 Departamento de Morfologia e Fisiologia Animal (DMFA), Universidade Federal Rural de Pernambuco. Av. Dom Manoel de Medeiros s/n, 52171-900 Dois Irmãos, Recife - PE, Brasil; pabyton.cadena@ufrpe.br

**Keywords:** marijuana, opioid, congenital anomalies, THC, CBD, morphine, codeine, zebrafish, birth defects

## Abstract

Marijuana and opioid addictions have increased alarmingly in recent decades, especially in the United States, posing threats to society. When the drug user is a pregnant mother, there is a serious risk to the developing baby. Congenital anomalies are associated with prenatal exposure to marijuana and opioids. Here, we summarize the current data on the prevalence of marijuana and opioid use among the people of the United States, particularly pregnant mothers. We also summarize the current zebrafish studies used to model and understand the effects of these drug exposures during development and to understand the behavioral changes after exposure. Zebrafish experiments recapitulate the drug effects seen in human addicts and the birth defects seen in human babies prenatally exposed to marijuana and opioids. Zebrafish show great potential as an easy and inexpensive model for screening compounds for their ability to mitigate the drug effects, which could lead to new therapeutics.

## 1. Introduction

The status of marijuana legislation for medical and recreational purposes has changed in many states of the United States in recent years. As of 2020, 33 states and the District of Columbia of the United States passed laws that broadly legalized marijuana in some form. Out of those 33 states, 11 states and the District of Columbia have adopted the legalization of recreational marijuana [1]. This change has altered societal views on the use of marijuana, making the attitude more permissive. The benefit of medical marijuana for the treatment of many illnesses, including chronic pain, nausea, and various neurological symptoms has become increasingly clear. Importantly, the Food and Drug Administration (FDA) approved Epidiolex (plant-based prescription cannabidiol (CBD)) for the treatment of people aged 2 years and older to treat drug-resistant epilepsies like Dravet syndrome and Lennox–Gastaut syndrome (LGS). No matter how beneficial its therapeutic properties are, all marijuana products have side effects. The gaining popularity, the potential benefits, and easy access to marijuana are making people more reluctant to consider its adverse effects. The report from the 2017 National Survey on Drug Use and Health (NSDUH) estimated 30.5 million people aged 12 or older used an illicit drug in the past 30 days (i.e., current use) [2]. Among these 30.5 million illicit drug users, 26.0 million were marijuana users, and 3.2 million misuse prescription pain relievers [2]. Notably, the potency of cannabis in marijuana products has shifted during the last two decades. A recent study has shown the overall increase in active ingredient content in the cannabis products, with greatly increased Δ9-tetrahydrocannabinol (THC: a psychoactive component from 3.4% in 1993 to 8.8% in 2008) relative to cannabidiol (a non-psychoactive component) [3,4]. These changes in use and potency of marijuana pose a high risk to the cannabis user [3].

Opioids are neuroactive drugs that are derived from the opium poppy (opiate) or synthesized in the laboratory (opioid). These drugs produce strong sedation, analgesia, and sleep. The use of opioids often begins with a prescription for acute pain treatment [5], but its long-term use can escalate to high doses, leading to addiction and overdose deaths [6]. Opioid overdoses have significantly increased in recent years. According to the World Health Organization, this increase is partly because of their frequent use as a pain medication to manage chronic non-cancer pain [7].

In the United States, opioid use either as prescription pain medications or as an illegal drug is widespread. There was an estimated 63,632 deaths due to drug overdose in the United States in 2016, out of which 19,413 deaths were associated with prescription opioids [7]. This was twice as high as the number in 2015 [7]. In 2018, ~67% of drug-related deaths (47,600 out of 70,237) involved opioid use [8]. The report from the 2017 NSDUH estimated that 11.4 million people aged 12 or older misused opioids in the past year, including 11.1 million pain reliever misusers and 886,000 heroin users. The majority of those people (62.6 percent) misused them to relieve physical pain [2].

## 2. Trends of Marijuana and Opioid Use during Pregnancy in Recent Decades

Increasingly, women of childbearing age use marijuana or opioids, which makes them very commonly used dependent substances during pregnancy in the United States after alcohol and tobacco [9,10]. Despite the fact that many states in the United States have adopted to legalize marijuana, none of the states have any regulation on its use during pregnancy. Cerdá M. et al. showed that the states with legal medical marijuana laws have significantly higher rates of marijuana use, abuse, and dependence compared to the states without such laws [11]. A nationwide study examining the prevalence of marijuana use among pregnant women using NSDUH data from 2007 to 2012 found out that 3.9% of pregnant women used marijuana in the previous month [12]. Another study used the NSDUH data from 2005 to 2014 and compared the marijuana use prevalence among married and unmarried pregnant women. This study showed that prenatal marijuana uses among unmarried women increased by 85% from 5.4% to 10% from 2005 to 2014, while the prevalence among married pregnant women remained mostly stable (1.5%) [13]. From 2009 through 2016, prenatal marijuana use in California increased from 4.2% to 7.1% [12,14]. Many women use marijuana to reduce morning sickness. This increasing trend of marijuana use by pregnant women is a great concern, especially when marijuana products contain high THC levels.

Opioid-containing medications are widely prescribed among reproductive-aged women with either private insurance or Medicaid [15]. In the United States, opioid overdose deaths among women increased more than five-fold between 1999 and 2010, and emergency department visits for misuse or abuse were even more frequent. For every opioid overdose death, there was about 30 emergency department visits [16]. Patrick et al. [17] found that 28% of around 112,000 pregnant women in the United States were prescribed opioid pain medication at least once during pregnancy, out of which 96.2% were short-acting opioid pain relievers. A study looking at the data from 2000 to 2007 with 1.1 million Medicaid-enrolled women from 46 U.S. states and Washington, DC found that one out of five women from that cohort of patients filled an opioid prescription during pregnancy [18]. The authors found that there was an increase in opioid prescriptions, especially codeine and hydrocodone, from 18.5% in 2000 to 22.8% in 2007 [18]. They also found a substantial regional variation in opioid prescription use during pregnancy ranging between 9.5% and 41.6% across the states. Another study of 534,500 women with completed pregnancies between 2005 and 2011 showed that 14.4% had an opioid prescription dispensed at some point during pregnancy. Of these, 2.2% had opioids dispensed three or more times during pregnancy [19]. This study, however, observed that there was a decline in opioid prescriptions to pregnant women from 14.9% to 12.9% between 2005 to 2011 [19]. A study done by the Centers for Disease Control and Prevention (CDC) estimated the proportion of privately insured and Medicaid-enrolled reproductive-aged women (15–44 years) who filled a prescription of opioid-containing medications from an outpatient pharmacy during 2008 to 2012, finding that approximately one in four privately insured and over one in three Medicaid-enrolled women filled a prescription for an opioid each year during that time. This study also found that an average of three opioids were prescribed for every four privately insured women and nearly two opioids were prescribed for each Medicaid-enrolled woman per year [15]. This increasing trend of opioid use by women of reproductive age is a great concern because 50% of pregnancies in the United States are unintended [15], and so, the exposure to the opioid during early prenatal development in unrecognized pregnancies is very likely.

These data on the marijuana and opioid use by reproductive-age women and pregnant mothers are alarming, which raises serious public health concerns as there is evidence of adverse pregnancy outcomes with prenatal marijuana or opioid exposure [20,21,22,23,24].

## 3. Patterns of Congenital Defects and Behavioral Changes Seen in Children Exposed to Marijuana in Utero

With the concern that legalization is making marijuana use more prevalent among pregnant women, Reece et. al. analyzed the overall patterns of birth defects in babies, without considering maternal age, in Colorado from 2000 to 2014 using data from the Colorado Responds to Children with Special Needs, the NSDUH, and the Drug Enforcement Agency [25]. This study identified multiple congenital defects that rose 5 to 37 times faster than the birth rate (3.3%) to generate in excess of 11,753 (22%) major anomalies. Those defects include cardiovascular (atrial septal defect, ventricular septal defect, patent ductus arteriosus, and others), spina bifida, microcephalus, Down’s syndrome, central nervous system, genitourinary, respiratory, chromosomal, and musculoskeletal defects [25]. They also showed that cannabis was the only drug whose use grew during that period, while other drugs like pain relievers, cocaine, alcohol, and tobacco did not [25]. This study indicates a connection between congenital defects and marijuana use among Coloradans. However, in a previous study done by Marleen et. al. looking at the babies born with and without major congenital malformations between 1997 to 2003 compared with in utero illicit drug exposure, the investigators did not find associations between birth defects and mother’s drug use (including marijuana) during pregnancy. They interviewed 15,208 mothers and examined 20 eligible categories of congenital malformations. Five percent of those mothers reported use of illicit drugs during pregnancy. However, they found the association between periconceptional cannabis use with an increased risk of anencephaly [26]. Other studies found links between in utero marijuana exposure to visual problem solving, visual-motor coordination, visual analysis, attention deficit, and behavioral problems [27,28]. Prenatal marijuana exposure was shown to be a significant predictor of marijuana use by adolescents [29].

## 4. Patterns of Congenital Defects and Behavioral Changes Seen after Prenatal Exposure to Opioid in Humans

The effect of prenatal exposure to opioids has been studied in recent years. Broussard et al. [30] described a correlation between opioid analgesic treatment in early pregnancy and certain birth defects, such as congenital heart disease, spina bifida, hydrocephaly, glaucoma, anterior chamber eye defects, and gastroschisis. Yazdy et al. [31] reported a two-fold increase risk of neural tube defects, including spina bifida, with maternal periconceptional opioid use. Minnes et al. [32] reviewed potential impacts on the development of the central nervous system and cognitive function associated with prenatal exposure to opiates and described less rhythmic swallowing, strabismus, possible cognitive delay, and defects in cognitive function. Moreover, autonomic dysregulation and nystagmus were associated with prenatal opiate exposed children [33]. Lind et al. [34] reported heart malformations as the most frequently reported malformations, followed by spina bifida and clubfoot in their systematic review of congenital defects related to maternal opioid use during pregnancy. However, the authors were concerned about the quality of the papers reviewed because of the lack of randomized control trials or other weaknesses found in the pregnancy literature on most medications. Maternal age was not taken into account in any of these studies.

Prenatal exposure to opiates leads to changes in behavior in babies. The prenatally opioid exposed neonate experiences opioid withdrawal symptoms because of the sudden discontinuation of the opioid after birth. Neonatal abstinence syndrome (NAS) is a postnatal drug abstinence syndrome characterized by a wide range of signs and symptoms including irritability, tremors, hypertonia, feeding intolerance, emesis, watery stools, seizures, and respiratory distress [17,23]. NAS-related withdrawal symptoms were recorded in 60 to 80 percent of newborns who were exposed to opioids such as heroin and methadone in utero [17]. The CDC reported diagnosis of seven NAS cases every 1000 newborn hospitalizations nationwide in 2016, which is three-fold higher than in 2008 (2.2 out of 1000 newborn) [35,36]. This is equivalent to 80 newborns with NAS every day [35,36]. In 1998, Bunikowski et al. reported NAS in the majority of drug-exposed infants during their first days of life. The researchers also found that children who are prenatally exposed to opiates had a mild developmental delay in the psychomotor system [37]. Minnes et al. [32] reviewed literature on potential impacts of prenatal opiate exposure and found a clear association between opiate exposure and anxiety, aggression, feelings of rejection, disruptive or inattentive behavior in babies. Recently, Conradt et al. reviewed the current literature to determine the short- and long-term neurodevelopmental outcomes of children with prenatal opioid exposure [38]. They also observed the symptoms in neonates as stated above. In addition, they showed that prenatal methadone or heroin exposure was associated with impaired mental, language, neuromotor, and psychomotor development. However, some of the studies they reviewed failed to account for important confounding variables or include a control group of unexposed children [38]. Lower IQ scores, differences in neurologic performance and language performance during elementary school education was also reported in the children exposed to opioids in utero. An increase in attention deficit hyperactivity disorder was observed in the children exposed to heroin in utero [33]. Children exposed to opioids prenatally also showed significantly lower visual–motor and perceptual performance scores [38].

These associations between in utero drug exposure and birth defects underscore the importance of understanding the mechanisms by which different opioids and marijuana ingredients cause birth defects. Different animal models are used to study the connections and mechanisms of opioid or marijuana use and specific birth defects [39].

## 5. Zebrafish: A Model System to Study the Effect of Marijuana and Opioid on Development and Behavior

Zebrafish is an outstanding model system to study vertebrate development. It is increasingly becoming a popular model to study the effects of exposure to different compounds during development because of its various advantages compared to the avian or mammalian systems [40,41,42,43,44,45,46]. It is easy to expose externally fertilized embryos to different drugs and to monitor their effects on development in transparent embryos. The developmental processes, genes, and regulatory networks are highly conserved between zebrafish and human. The zebrafish has been extensively used to study congenital defects including heart, eye, and neurodevelopmental defects caused by alcohol exposure [47,48,49,50,51,52,53,54] or the exposure to different environmental toxicants during development [43]. The zebrafish studies can dissect cellular and molecular changes during early embryogenesis (gastrulation) due to alcohol exposure [49,51]. Although the zebrafish brain is much simpler than a mammalian brain, it has a homologous organization, similar cellular morphology, and neurochemistry with mammals. Zebrafish larvae and adults have increasingly been used to study the nervous system, brain disorders, including disorders due to abuse of drugs seen in complex behaviors. Kalueff et al. wrote a comprehensive review showing striking similarities between zebrafish and mammalian brain biology and neurochemistry [55]. Zebrafish display anxiety, stress, and mood disorders, like other vertebrates [55,56]. Behaviors such as environment exploration, preference for light/dark environments, bottom-dwelling, peripheral preference, freezing/immobility, and irregular movements are used to measure anxiety, stress, and mood [57]. Behavioral phenotypes observed in zebrafish are strikingly similar to those observed in humans and rodents [55,57]. Thus, major brain disorders can be modeled in zebrafish in a cost-effective and simple way. As with any animal models in biomedicine, zebrafish have limitations and cannot fully recapitulate complex human brain disorders. Furthermore, some brain regions are less evolved than in mammals (e.g., the cerebral cortex) [57]. While some features, like neural physiology complexity, have been studied in less detail in zebrafish than mammalian models; the simplicity of the zebrafish model combined with genetic and genomic knowledge makes it attractive and useful [57].

### 5.1. Zebrafish Endocannabinoid Biology: A Gene-Level Comparison with Human

The human endocannabinoid system (eCBs) is composed of a set of cannabinoid receptors (mainly CNR1 and CNR2), endogenous cannabinoid ligands (2-arachidonoylglycerol, 2-AG; anandamide, AEA), different enzymes responsible for biosynthesis of cannabinoids (e.g., diacylglycerol lipase, DAGLa and DAGLb; ab-hydrolase domain containing 4, ABHD4; N-acyl phosphatidylethanolamine phospholipase D, NAPE-PLD; Glycerophosphodiester phosphodiesterase 1, GDE1) and degradation of cannabinoids (e.g., ab-hydrolase domain containing 6b, ABHD6B; fatty acid amide hydrolase families, FAAH and FAAH2A; monoglyceride lipase, MGLL) [58,59]. Unlike invertebrates, the eCB system is highly conserved between zebrafish and mammals, including humans. The zebrafish contain orthologs of all human cannabinoid signaling genes except N-acylethanolamine acid amidase gene [40,58,59]. Another difference is that zebrafish have two homologs for each of the four human genes FAAH2, PTGS2, TRPA1, and PPARA. Zebrafish Cnr1 and Cnr2 share 75% and 46% identity with their human counterparts [40,58].

An extensive body of research on the expression and the functions of zebrafish eCB genes are summarized in recent reviews [40,45,58,59]. During development, the expression of zebrafish cannabinoid receptor 1 (*cnr1*) was first detected in the pre-optic area as early as 1 day post fertilization (dpf). At the later larval stage, *cnr1* expression was detected in different parts of the brain, including the telencephalon, hypothalamus, tegmentum, and anterior hindbrain. Examining the expression in adult zebrafish brain showed a similar expression pattern [58,60,61,62]. Francesca et. al. detected high level cannabinoid receptor 2 (*cnr2*) mRNA expression as early as 4 hours post fertilization (hpf) [58]. The expression was reduced by 12 hpf and then went up again. In adults, *cnr2* mRNA was detected in gills, heart, intestine, muscle, spleen, and central nervous system [63].

The mRNAs encoding *dagla* and *daglb*, enzymes involved in the biosynthesis of the most abundant endocannabinoid 2-AG, were first detected during the early cleavage period suggesting that these mRNAs were maternally transmitted [58]. However, *dagla* and *daglb* were not detected during gastrulation and somitogenesis but detected again after 1 dpf [58]. In situ hybridization showed the expression of *dagla* throughout the anterior and posterior hindbrain at 2 dpf [62]. The mRNA expression of *nape-pld, abdh4,* and *gde1,* gene encoding enzymes involved in the synthesis of AEA, showed similar pattern as *dagl* genes. These genes also appeared to be maternally transmitted and were degraded during the pre-gastrulation period [58]. Francesca et. al. examined the expression until 4 dpf and showed that the zygotic expression begins around somitogenesis, which persisted at all time points that they examined [58].

The mRNAs encoding 2-AG degrading enzymes, *mgll*, and *abhd6a* were detected at low levels during early embryogenesis through organogenesis [58]. The 2-AG degrading enzyme *abhd12* was maternally transmitted, and its expression was high during early embryogenesis. The expression of *abhd12* was detected at all stages tested, until 4 dpf [58]. The mRNAs encoding proteins Faah and Faah2a, enzymes that degrade AEA, were detected at 1 hpf embryos, suggesting that those genes were maternally transmitted [58]. Zygotic transcripts were detected from 1 dpf until 4 dpf, the stages tested [58]. Table 1 summarizes the expression patterns of the zebrafish endocannabinoid signaling genes.

Receptor-binding assays were done to examine if typical cannabinoid ligands for mammalian receptors can similarly bind to the zebrafish eCB receptors [70,71]. The study using the zebrafish brain homogenates and radiolabelled cannabinoid showed that a few typical cannabinoids did not fully recognize the cannabinoid-binding sites in zebrafish brain, but HU-210, WIN55212-2 and CP55940 bind to zebrafish eCB receptors very well. The affinity of the cannabinoids was determined as HU-210 > WIN55212-2 > CP55940 >anandamide [58,59,70]. Connors et al. showed that radiolabeled WIN55212-2 binds to the hypothalamus, optic tectum, and telencephalon targets in adult zebrafish brain sections [58,71].

The interactions of zebrafish receptors with those synthetic cannabinoids and the similarity in expression of zebrafish eCB genes with mammalian cannabinoid (CB) genes suggest that zebrafish CB signaling may serve the same function as mammalian CB signaling [59], and hence, zebrafish are a potentially useful model to study the effects of cannabinoid abuse on development and behavior.

### 5.2. Zebrafish Opioid Biology—Why the Zebrafish Is a Useful System to Study the Effects of Opioids

The human opioid signaling system consists of primarily three classical opioid receptors: the mu-opioid receptor (MORP), the kappa-opioid receptor (KORP), and the delta-opioid receptor (DORP). The fourth opioid receptor is nociception receptor (NOP). The NOP system is considered to be a part of the opioid system, although it produces antiopioid actions depending on the region in which it is expressed in the brain. These receptors are activated by endogenous ligands ß-endorphin (MORP ligand), dynorphin (KORP ligand), enkephalin and deltorphin (DORP ligand), nociceptin/orphanin FQ (N/OFQ) (NOP ligand) that result in physiological effects including analgesia, dysphoria, water diuresis, and antipruritic effects [40,45]. These ligands are derived from precursor compounds that are produced by the genes POMC, PENK, PDYN, and PNOC.

All four opioid receptors, the mu-opioid receptor, the kappa-opioid receptor, the delta-opioid receptor, and the nociception receptors are present in zebrafish [40,45]. Zebrafish have single homologs (*oprm1, oprk1, oprl1*) for three of the human opioid receptor genes, but the gene encoding delta-opioid receptor has a pair of homologs *oprd1a* and *oprd1b* [40,45]. Zebrafish and human opioid receptor genes and proteins share an average of 72% (range: 64%–77%) and of 87% (range: 77%–100%) identity, respectively [40,45]. Zebrafish has single homolog for *pdyn* but two copies of *pomc*, *penk,* and *pnoc* homologs [40,45]. The expression analyses of zebrafish opioid receptor genes showed that all opioid receptor genes are expressed during the first day of development, although *opkr1* expression is very low [45,72]. Robust expression of *oprm1* starts as early as 3 hpf, but high levels of *oprd1a* and *oprd1b* were detected first at 22 hpf [45,72]. The levels of expression for these receptors vary during different developmental stages, peaking at different times. The transcripts encoding NOP *(oprl1)* was detected at 0.5 hpf, which suggests that it is deposited maternally. Its expression was detected throughout embryonic development [73]. The expression of *oprk1* was first detected at 48 hpf [45].

In situ hybridization detected wide expressions of *opmr1* and *opd1b* in multiple CNS structures, including the telencephalon, epiphysis, diencephalon, midbrain, isthmus, cerebellum, pretectum, and hindbrain at 24 hpf, although the *opd1b* expression was weaker than *opmr1* [45,72]. Additional expression of *opd1b* was detected in the myotomes and spinal cord [72]. The expression patterns of these genes were more defined and at different CNS structures at 48 hpf [45,72]. The localization of *oprd1a* was detected in the telencephalon, epiphysis, pretectum, and cerebellum at 24 hpf and the hindbrain, spinal cord, and tegmentum at 30–36 hpf [45,72]. Table 2 summarizes the expression patterns of the zebrafish opioid signaling genes.

In general, the opioid receptors in zebrafish have analogous, conserved functions and pharmacological properties when compared to mammalian proteins, but there are differences in ligand selectivity between species caused by evolutionary changes of their receptor sequences [82].

### 5.3. Zebrafish Studies on Effects of Embryonic Cannabinoid Exposure on Development

Given the embryonic expression of genes involved in eCB signaling including cannabinoid receptors, it is imperative to understand how modulation of the endocannabinoid system during embryonic development affects the morphology and the behavior of the fish. In the last three decades, several studies were done using zebrafish mutants and transgenic lines (deficient or overexpressed receptors), morpholinos, and the treatment of agonists or antagonists of the receptors. Those studies help decipher the roles of cannabinoid signaling in addiction, anxiety, immune system, energy homeostasis, food intake, learning and memory, and embryo development. The results of those studies were summarized in recent reviews [40,58]. Here we are emphasizing the most recent zebrafish studies that examined the effects of embryonic cannabinoid exposure on development, which shed light on the consequences of disrupting normal eCB signaling on the formation of different cell types, tissues, and organs. Endogenous cannabinoids (2-AG and AEA), synthetic cannabinoids, and cannabinoid antagonists were used in these studies (Table 3).

Alteration of CB signaling during embryonic development has profound effects on the survival and hatching of zebrafish embryos. Ahmed et al. exposed the embryos to either psychoactive or non-psychoactive ingredients of marijuana (THC: 2–10 mg/L diluted from a stock solution of 1.0 mg/mL in methanol or CBD: 1–4 mg/L diluted from a stock solution of 1.0 mg/mL in methanol) during gastrulation between 5.25 hpf to 10.75 hpf [83]. Brief exposure to THC or CBD during gastrulation, only for a period of 5$\frac{1}{2}$ h, significantly reduced the hatching rate of the larvae at 3 dpf. CBD was more potent in decreasing hatching than THC. CBD reduced the hatching to zero at the highest concentration tested [83]. The same treatment regimens of THC or CBD significantly reduced the survival rate of the larvae in a dose-dependent way. Like its effect on hatching, CBD exposure exerted more severe effects on survival, causing the death of more larvae [83]. Willett and her group employed waterborne exposure to CBD and THC by dissolving THC (0.3–5.0 mg/L) or CBD (0.07–1.25 mg/L) in 0.05% DMSO and validated their assay by measuring the water concentration and tissue bio-concentration of the drugs [84]. The actual THC concentrations in water at the start of exposure were between 64% and 88% of the expected concentrations, which declined to 16–32% of the initial concentration after 96 h of exposure, and actual CBD concentrations were only 33–40% of the expected concentrations that decreased to 0–3% of the initial concentration after 96 h of exposure [84]. At the end of the exposure, they measured the concentrations of the drugs in larvae and detected 0.28 mg/g of THC or 79 mg/g of CBD after a nominal 0.3 mg/mL of THC or CBD exposure. The embryos treated with higher concentrations of either THC or CBD from 2 to 96 hpf produced embryonic lethality [84]. The lethality by Δ9-THC exposure was observed and published as early as 1975, showing that exposure to 5 ppm or 10 ppm Δ9-THC from late blastula stage to 24 hpf led to the death of the larvae after 24 h [86].

The effects of specific CNR1 ligands showed different results to THC and CBD exposure. THC and CBD interact with both CNR1 and CNR2 receptors (THC binds to both types of receptors and CBD modifies the receptors’ ability to bind to cannabinoids). Continuous exposure to different CNR1 receptor ligands WIN55,212-2 (1 nM-1 µM), AM251 (100 nM-5 µM) or rimonabant (SR141716A:1 nM-1 µM) at various concentrations from 0–72 hpf or 0–96 hpf, without the replacement of solution for the entire exposure period, did not impact hatching [88]. Although the highest concentration of WIN55,212-2 (1 µM; CNR1 agonist) caused complete embryonic lethality, lower concentrations had no impact on the survival of the embryos. Exposure to CNR1 antagonists AM251 or rimonabant also did not change the survival rate [88]. In another study, Migliarini et. al. showed that continuous exposure to AM251 at concentrations of 10 and 20 nM reduced hatching rates [89]. These studies suggest that modulating the cannabinoid system, specially CNR2 mediated cannabinoid signaling, even for a brief period during early development leads to lethality.

Short exposure to the THC (2–10 mg/L) or CBD (1–4 mg/L) during gastrulation causes many morphological changes in the embryos. The exposure caused deformities in the trunk including short body, curved tails, and blebbing at the tip of the tail. Higher exposure concentrations produced more severe deformities [83]. Waterborne exposure to THC (0.3–2.5 mg/L) or CBD (0.07–1.25 mg/L) from 2 to 96 hpf caused short and deformed body axis and bent distal trunk [84]. In fact, similar morphological anomalies were reported when embryos were exposed to 1–10 ppm Δ9-THC from the blastula stage until 24 hpf [86]. The morphology of the larvae was observed until 9 dpf. The lowest concentration of Δ9-THC (1 ppm) did not change the morphology of the larvae, but exposure to 2 ppm caused distal trunk anomalies that included a curved spine or bulbous-tipped tail [86]. On the other hand, continuous exposure to CNR1 antagonist AM251 (10 or 20 nM in ethanol) did not cause any distinguishable morphological changes [89]. These results indicate that cannabinoid signaling mediated by CNR2 receptor is active in the distal trunk during early development and blocking CNR1 receptor does not alter trunk morphology.

Early exposure to exogenous cannabinoids had negative effects on the heart rate. Exposure to THC (4–10 mg/L) during gastrulation from 5.25 to 10.75 hpf reduced the heart rate of larvae in a dose-dependent manner when examined at 2 dpf. The highest concentration of THC reduced the heart rate to 50% of the untreated control. Similar to THC, CBD exposure (1–4 mg/L CBD) for the same duration also reduced the heart rate of the larvae at 2 dpf, producing an even greater effect than that of THC [83]. Waterborne exposure to THC (0.3–2.5 mg/L) or CBD (0.07–1.25 mg/) from 2 to 96 hpf caused pericardial edema. This report also showed that CBD exposure was more potent in causing pericardial edema than THC, which exerted similar defects at a ten-times lower concentration than THC. The highest concentrations of both the drugs tested caused pericardial edema in all the embryos [84].

Chronic exposure to CNR1 agonist ACEA (6 mg/L suspended in ethanol) from 6 to 24 hpf induced microphthalmia, but a lower concentration of ACEA (3 mg/L) did not. However, when the embryos were treated with the combinations of 3 mg/L ACEA and 0.5% ethanol, the larvae had smaller eyes, suggesting a synergistic effect of CNR1 agonist and alcohol on interfering eye development [90]. Binge-like exposure of ACEA (3 and 12 mg/L) on eye development was examined by treating embryos during key developmental time points including the first hour of gastrulation (5.25–6.25 hpf), transition from gastrulation to neurulation (8–10 hpf) or during the formation of the five-vesicle brain (24–27 hpf). Higher concentrations of ACEA exposure at all the stages examined caused microphthalmia. Lower concentration (3 mg/L) of ACEA alone did not cause microphthalmia, but when treated with ethanol (1%), ACEA synergized with ethanol to induce microphthalmia. This study highlights the synergistic interaction of the CNR1 agonist and alcohol and shows how alcohol exposure exacerbates the defects caused by ACEA exposure [90]. Smaller eyes were also reported following waterborne exposure to CBD and THC [84]. These studies demonstrated the effect of prenatal cannabinoid exposure on eye development.

THC or CBD exposure during gastrulation from 5.25 to 10.75 hpf altered morphology and innervation patterns of primary and secondary motor neurons [83]. Exposure to CBD (3 mg/L) during gastrulation reduced the number of axonal branches of primary neurons in 2 dpf embryos, but THC exposure (6 mg/L) for a similar duration did not cause any change in primary neuron morphology. Both THC (6 mg/L) and CBD (3 mg/L) exposure exert severe effects on the secondary motor neurons compared with primary motor neurons. Dorsal, ventral, and lateral motor neuron branching patterns analyses using fluorescent labels showed undetectable dorsal branches and thinner ventral branches of secondary motor neurons in embryos treated with either 6 mg/L THC or 3 mg/L CBD. THC exposure did not alter lateral motor neuron branches, but CBD treatment significantly reduced those branches. Cannabinoid treatment altered synaptic activity at neuromuscular junctions (NMJs) and altered nicotinic acetylcholine receptor expression at NMJs [83]. Exposure to 6 mg/L THC during gastrulation, from 5.25 to 10.75 hpf slightly altered the axon diameter of Mauthner cells (M-cell), the neurons born during gastrulation and which are involved in escape response movements [85]. The muscle fibers of those larvae were slightly disorganized with subtle but significant changes in nicotinic acetylcholine receptor expression patterns [85].

A recent zebrafish study revealed the role of Cnr2 signaling on the production, expansion, and migration of embryonic hematopoietic stem cells [87]. By using different Cnr1 and Cnr2 signaling chemical modulators (Table 3) and genetic approaches, the investigators showed that Cnr2, but not Cnr1 regulates embryonic hematopoietic stem cell development. Cnr2-signaling optimizes the production, expansion, and migration of embryonic hematopoietic stem cells by modulating multiple downstream signaling pathways [87].

### 5.4. Zebrafish Studies Examining Effects of Embryonic Opioid Exposure on Development

Although the opioid system is active during early development and evidence shows the association of in utero opioid exposure and congenital defects in humans, there are only a handful of zebrafish studies aiming to understand the association between opioid exposure and birth defects. Sanchez-Simon et al. treated zebrafish embryos with 1, 10, and 100 nM of morphine during three different developmental periods: 5–24, 5–48 and 5–72 hpf to determine the effects of embryonic morphine exposure on neuronal fate [91]. They reported that 100 nM caused severe malformation and death in the embryos, while 1 nM did not produce measurable effects. Chronic exposure to 10 nM of morphine from 5–24 or 5–48 hpf enhanced cell proliferation and either induced or suppressed neuronal differentiation depending on neuronal populations. This study also showed that morphine protected motor and Pax-6-positive neurons against glutamate-induced damage. Interestingly, morphine exposure altered the expression of the opioid receptor genes [91]. The authors measured the concentration of morphine in the embryos after chronic exposure (5–24 or 5–48 hpf) and reported that only ~ 5% of the treatment concentration (10 nm) was present in the embryos [91]. In a later study, the group showed that morphine exposure caused nestin overexpression and delayed neural stem cell differentiation [92]. Microarray analyses after chronic exposure to 10 nM morphine from 5 to 24 hpf identified alteration of several genes associated with mu-opioid receptor expression and genes involved in neuronal development, CNS patterning processes, neuronal differentiation, dopaminergic neurotransmission, the serotonergic signaling pathway, and glutamatergic neurotransmission [93]. Rodriguez and her group showed that embryonic exposure to nociceptin altered the temporal and spatial expression pattern of NOP transcripts at 24 and 48 hpf [73]. Since the opioid system is active during zebrafish embryonic development, the advantages of the zebrafish model system can be exploited to understand prenatal opioid exposure effects.

### 5.5. Zebrafish Studies Examine Behavioral Defects Associated to Embryonic Exposure to Marijuana or Opioid

Many studies showed that the zebrafish behavioral repertoire changes after they have been exposed to psychoactive drugs, and those effects can be assayed using various techniques. Basnet et al. [56] nicely outlined the zebrafish behavior repertoire that can be used in neuropharmacology and reviewed the current literature on the utility of zebrafish larvae as a behavioral model in neuropharmacology. Zebrafish larvae show key behaviors like stress and anxiety. Zebrafish larvae are sensitive to different stimuli such as touch, olfaction, chemosensing, audition, heat, and vision. The zebrafish behavioral assay repertoire includes thigmotaxis, avoidance, startle response, optokinetic response, optomotor response, habituation, prey capture, response to predators, sleep/wake behavior, and locomotor response [56,94,95]. Zebrafish used as models of drug addiction (self-administration and conditioned place preference), withdrawal, anxiety-like behavior, pain, and cognition after cannabinoid and opioid exposure was recently reviewed [40], and those studies demonstrate the utility of zebrafish in studying human physiological conditions after drug exposure. Zebrafish could also be used to screen compounds with the potential to attenuate the drug effects, like opioid withdrawal syndrome symptoms. Khor et. al. modeled a morphine-withdrawal situation by treating adult fish continuously with 1.5 mg/L morphine for two weeks followed by withdrawal for 24 h, and the zebrafish displayed decreased exploratory behavior, increased erratic movement, elevated cortisol levels, indicating anxiety-related responses and stress [96]. Mitragynine is an alkaloid found in leaves of *Mitragyna speciosa*, a plant widely used by opiate addicts to mitigate the harshness of drug withdrawal. Exposure to mitragynine during the withdrawal phase attenuated majority of the stress-related swimming behaviors and lowered the whole-body cortisol level [96]. The authors also showed that mitragynine reduced the mRNA expression of corticotropin-releasing factor receptors and prodynorphin in zebrafish brain during the morphine withdrawal phase [96]. This study shows for the first time a possible link between mitragynine’s ability to attenuate anxiety during opiate withdrawal and provided evidence for the utilization of the zebrafish to screen compounds for their ability to mitigate drug withdrawal effects. Together, these studies demonstrate the effectiveness of zebrafish as a model to study drug addiction and withdrawal.

## 6. Summary

**Figure 1:**
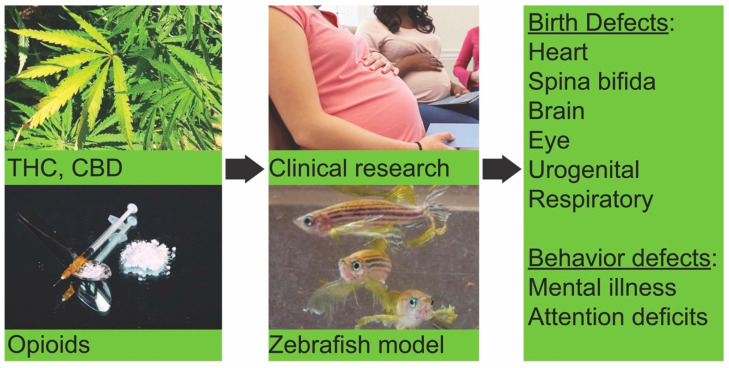
Schematic representations of drug use/abuse during pregnancy and birth outcomes. In utero exposure to Δ9-tetrahydrocannabinol (THC), cannabidiol (CBD) or opioids leads to congenital malformations and behavioral changes in babies. Exposure to cannabinoids or opioids during zebrafish development produced similar defects in embryos.

Marijuana and opioid abuse are serious global health problems, which are more serious in the United States than most other countries, causing more than half a million deaths in the last two decades. The use of these drugs by pregnant and reproductive-age women has significantly increased in recent years. There is strong evidence for the association of prenatal drug exposure and congenital anomalies (Figure 1), which raises additional serious public health concerns. To combat the growing opioid overdose crisis, it was declared to be a public health emergency by the White House in 2017 [97]. The CDC outlined different focus areas to fight the drug abuse epidemic, and one of these is to advance research to better understand the epidemic and identify effective strategies to prevent it [8]. It will be important to better understand whether different cannabinoids and opioids exposure lead to adverse outcomes in babies, which would magnify the consequences of drug use in society. A challenge of evaluating drug exposure and birth defects is the potential contribution of other confounding factors such as maternal age, prenatal care, prenatal vitamins, diet, and exposure to other teratogens such as alcohol. For the studies related to developmental delay and cognitive function in babies, it is difficult to control for environment, nutrition, and socioeconomic factors. Using animal models, the direct effects of the drugs can be determined independent of other confounding factors.

Zebrafish provide a rapid and inexpensive platform to study the effects of drug exposure during development. Zebrafish studies showed various developmental defects caused by embryonic exposure to marijuana components (Figure 1). Those studies also indicated the differential sensitivity of the exogenous cannabinoids. Although the number of zebrafish studies examining the effects of different opioids on development is small, the existing evidence also showed developmental defects in fish. Zebrafish studies showed behavioral effects similar to human drug addiction and withdrawal symptoms. Although the human behavior response can never be completely replicated in the zebrafish, the fish experiments suggest that many drug-induced human and zebrafish phenotypes share common genetic and physiological factors. Recently, high-throughput zebrafish behavioral screens identified a novel group of antipsychotics, finazines, and their target, the sigma non-opioid intracellular receptor 1 [98,99,100]. These studies showed the utility of zebrafish in the discovery of new therapies for psychiatric disorders. Overall, zebrafish represents an exciting and emerging model to study the effects of drug exposure on development and could be a useful alternative vertebrate model for translational studies.

## Figures and Tables

**Table 1:** Expression patterns of the zebrafish endocannabinoid signaling genes.

Gene Name	Maternal Deposition (Detected by RT-PCR)	First Zygotic Expression (Detected by RT-PCR)	Expressions in the Tissue (Detected by in situ Hybridization)
cannabinoid receptor 1 *(cnr1*)	yes [58]	1 dpf [58]	pre-optic area at 30 hpf; telencephalon, diencephalon, and midbrain at 50 hpf; olfactory bulb, midbrain, endoderm and liver at 72 and 96 hpf
cannabinoid receptor 2 *(cnr2*)	yes [58]	4 hpf * [58]	developing central nervous system at 24, and 48 hpf [64]; developing central nervous system, endoderm and liver at 72 and 96 hpf [64]
diacylglycerol lipase 1a *(dagla*)	yes (low expression) [58]	4 hpf * [58]	whole organism at 5–9 somite [65]; cranial ganglion, hindbrain, hypothalamus, midbrain, tegmentum, telencephalon at 48 hpf [62]
diacylglycerol lipase 1b *(daglb*)	yes (high expression) [58]	4 hpf * [58]	no in situ data at the embryo/larval stage
N-acyl phosphatidyl-ethanolamine phospholipase d *(napepld*)	yes (low expression) [58]	4 hpf * [58]	whole organism from 5 somite to 4 dpf [65]
ab-hydrolase domain containing 4 *(abhd4)*	yes (high expression) [58]	4 hpf * [58]	whole organism from 1 cell to 4 dpf [65,66],
Glycerophospho-diester phosphodiesterase 1 *(gde1*)	yes (high expression) [58]	4 hpf * [58]	basal level expression throughout the body at 1 cell to pec-fin stage [66].
ab-hydrolase domain containing 6a *(abhd6a*)	yes (low expression) [58]	24 hpf [58]	no in situ data available
ab-hydrolase domain containing 6b *(abhd6b*)	yes (moderate expression) [57]	72 hpf [58]	no in situ data available
ab-hydrolase domain containing 12 *(abhd12*)	yes (high expression) [57]	4 hpf [58]	brain, gill, neuromast at 5dpf [67]
fatty acid amide hydrolase family *(faah)*	yes [57]	24 hpf [58]	intestinal bulb, liver at long-pec to 4 dpf [68]
fatty acid amide hydrolase family 2a *(faah2a)*	yes [58]	24 hpf [58]	intestinal bulb, liver at long-pec to 4 dpf [66,68]
fatty acid amide hydrolase family 2a (*faah2a)*	yes [68]	3 hpf	intestinal bulb, liver at long-pec to 4 dpf [68]
monoglyceride lipase *(mgll)*	no [58]	4 hpf [58]	whole organism at 5–9 somite [65] brain, eye, pectoral fin, pronephric duct, pharynx at stages 26 somite to long-pec [65,69].

* the expression level varies at different developmental stages and peaks at different times.

**Table 2:** Expression patterns of the zebrafish opioid signaling genes.

Gene Name	Maternal Deposition (Detected by RT-PCR)	First Zygotic Expression (Detected by RT-PCR)	Expressions in the Tissue (Detected by in situ Hybridization)
mu-opioid receptor *(oprm1)*	yes [72]	3 hpf * [45,72]	telencephalon, epiphysis, diencephalon, midbrain, isthmus, cerebellum, pretectum, and hindbrain at 24 hpf; tegmentum, hypophysis, otic vesicle, and pectoral flipper at 48 hpf [45,72]
kappa-opioid receptor *(oprk1)*	yes (low expression) [65]	3 hpf [45,72]	no in situ data of the developing stages available
delta-opioid receptor *(oprd1a)*	yes (moderate expression) [65]	3 hpf [45,72]	whole organism at tail-bud stage [74]; telencephalon, epiphysis, pretectum, and cerebellum at 24 hpf; hindbrain, spinal cord, and tegmentum at 30–36 hpf [45,72]
delta-opioid receptor *(oprd1b)*	Yes (moderate expression) [65]	3 hpf [45,72]	whole organism at tail-bud stage [74]; telencephalon, epiphysis, diencephalon, midbrain, isthmus, cerebellum, pretectum, hindbrain, myotomes and spinal cord at 24 hpf [45,72]
nociception receptor *(oprl1*)	yes [73]	3 hpf [73]	diencephalon, hindbrain, midbrain, pretectum, telencephalon at 24 hpf [72]
prodynorphin *(pdyn)*	no data available	no data available	hypothalamus, lateral region at 2 dpfhindbrain, neuron at 5 dpf
proopiomelanocortin a *(pomca)*	yes [75]	shield [75]	whole organism at 64 cell [76]; Pituitary, hypothalamus at 2–5 dpf [77,78]
proopiomelanocortin b *(pomcb)*	no data available	no data available	preoptic area at 3 dpf [78]
proenkephalin a *(penka)*	no data available	no data available	diencephalon, epiphysis, dorsal telencephalon subpopulation of dorsal spinal cord neurons at 22–25 somite, 30–42 hpf [66]; central nervous system, retina at 5 dpf [66]
proenkephalin b *(penkb)*	no data available	no data available	dorsal posterior midbrain, diencephalon, spinal cord, posterior pronephric ducts at 22–25 somite, 30–42 hpf, and additionally hindbrain at 60 hpf [66]
prepronociceptin a *(pnoca)*	no data available	no data available	alpha pancreatic cells at 30 hpf [79], posterior pancreatic bud at 2 dpf [79]
prepronociceptin b *(pnocb)*	no data available	no data available	neurogenic field, preplacodal ectoderm at bud to 1–4 somites [80]; brain at 5 dpf [81]

* the expression level varies at different developmental stages and peaks at different times.

**Table 3:** Cannabinoid signaling modulators used in zebrafish studies and the treatment period during development.

Compound	Target	Concentration	Treatment Period
∆9-Tetrahydro-cannabinol (THC)	CNR1/2 agonist	2–10 mg/L [83] 1–16 µM (0.3–5.0 mg/L) [84] 6 mg/L [85] 1 ppm–10 ppm [86]	5.25–10.75 hpf 2–96 hpf 5.25–10.75 hpf blastula-24 hpf
Cannabidiol (CBD)	CNR1/2 agonist	1–4 mg/L [83] 0.25–4 µM (0.07–1.25 mg/L) [84]	5.25–10.75 hpf 2–96 hpf
2-Arachidonoyl-glycerol (2-AG)	Endogenous CB	5 µM [87]	18–24, 30–36, or 30–96 hpf
Anandamide (AEA)	Endogenous CB	5 µM [87]	18–24, 30–36, or 30–96 hpf
O2545	CNR1/2 agonist	5 µM [87]	12–30 hpf
Arachidonyl-2’-chloroethylamide (ACEA)	CNR1 agonist	5 µM [87]	5.25–6.25, 8–10, 24–27 hpf
AM1241	CNR2 agonist	5–10 µM [87]	18–24, 30–36, 30–96 hpf
JWH015	CNR2 agonist	JW 5–10 µM [87]	12–30 hpf
WIN55,212-2	CNR1 agonist	1 nM–1 µM [88]	0–72, 0–96 hpf
AM251	CNR1 antagonist	100 nM–5 µM [88]; 10 nM, 20 nM [89]	0–72, 0–96 hpf
Rimonabant (SR141716A)	CNR1 antagonist	1 nM–1 µM [88]	0–72, 0–96 hpf
AM630	CNR2 antagonist	5–10 μM [87]	18–24, 30–38, 30–48, 30–96 hpf
